# Ectopic pregnancies: laparoscopic versus vNOTES approach. Surgical and obstetric outcomes

**DOI:** 10.1007/s00404-025-08098-0

**Published:** 2025-07-02

**Authors:** Anne-Sophie Vertongen, Andrea Stuart, Kristin Stuhr Olsson, Karin Källén, Jan Baekelandt

**Affiliations:** 1 Department of Obstetrics and Gynaecology, AZ Voorkempen, Malle, Belgium; 2 Department of Obstetrics and Gynecology, Helsingborg Central Hospital, Helsingborg, Sweden; 3 Institution of Clinical Sciences, Department of Obstetrics and Gynecology, Lund University, Lund, Sweden; 4 Centre for Reproductive Epidemiology, Tornblad Institute, Lund University, Lund, Sweden; 5 Department of Obstetrics and Gynaecology, Imelda Hospital, Bonheiden, Belgium

**Keywords:** vNOTES, Laparoscopy, Ectopic pregnancy, Obstetrical outcome

## Abstract

**Introduction:**

Extrauterine pregnancy (EP), represents a significant challenge in reproductive medicine, manifesting in approximately 2% of all pregnancies, primarily implanting within the fallopian tubes (95%). Surgery remains a cornerstone in the therapeutic options for ectopic pregnancies. The most common surgical approach at the moment is laparoscopy. However a relatively new surgical technique, vaginal Natural Orifice Transluminal Endoscopic Surgery (vNOTES) has evolved which is a vaginal entry to the abdomen in conjunction with endoscopy via the vagina. In this study we compare the surgical and obstetrical outcomes of these two techniques.

**Results:**

The operation time using the vNOTES approach was significantly shorter when compared to a laparoscopic approach, mean surgical time was 35 versus 47 min (p < 0.001). Both techniques had a very low complication and postoperative infection rate. Duration of hospital stay was not significantly lower in the vNOTES group but a trend was seen (p = 0.06). No significant differences were observed in the obstetric outcomes.

**Conclusion:**

This study shows that the vNOTES approach is a good and safe technique to operate ectopic pregnancies and can provide shorter operating time and duration of hospitalisation. Early data shows promising results on safety for future pregnancies but further research is warranted.

## What does this study adds to the clinical work

**Table:** 

vNOTES approach to operate ectopic pregnancies shows quicker surgical time than a laparoscopic approach. No difference in obstetrical outcome or perineal tears in subsequent deliveries was found between woman who previously were operated for an ectopic pregnancy with vNOTES vs laparoscopic approach.	

## Introduction

Extrauterine pregnancy (EP), represents a significant challenge in reproductive medicine [1], manifesting in approximately 2% of all pregnancies, primarily implanting within the fallopian tubes (95%) [2, 3]. Recognised risk factors are prior ectopic pregnancies, pelvic inflammatory disease (PID), tubal surgeries, cigarette smoking, pregnancy with an intrauterine device (IUD), age > 35 years and assisted reproductive technologies, contributing to the growing incidence of this condition [3–6].

Treatment options for tubal ectopic pregnancies include: watchful waiting, treatment with methotrexate or surgery [5, 6]. Watchful waiting and methotrexate are only safe when there are no contra-indications.

Surgery remains a cornerstone in the therapeutic options for ectopic pregnancies. In cases marked by severe tubal damage or rupture, salpingectomy is the recommended option. Conversely, salpingostomy, preserving the fallopian tube whilst excising the ectopic pregnancy, stands as an alternative approach when feasible [1, 5].

The most common surgical approach at the moment is laparoscopy. However a relatively new surgical technique, vaginal Natural Orifice Transluminal Endoscopic Surgery (vNOTES) has evolved which is a vaginal entry to the abdomen in conjunction with endoscopy via the vagina [7–13]. vNOTES has shown to reduce surgical time and postoperative pain for hysterectomy and adnexal surgery compared to a laparoscopic approach [7, 8, 11] and also has been proven safe for future pregnancies in a small study [14].

The aim with this study is to compare surgical outcome and future pregnancy outcome after standard laparoscopic technique compared to the vNOTES technique for the surgical treatment of ectopic pregnancies.

## Materials and methods

### Study design and participants

A retrospective descriptive cohort study of all patients treated surgically for an ectopic pregnancy between 01/01/2013 and 31/01/2024 at Imelda hospital in Bonheiden, Belgium were included. The ethical committee of Imelda hospital approved the collection of data from the patient’s files.

For treating ectopic pregnancies a 2 cm posterior colpotomy was made for access to the abdomen. An Alexis ring, (Gelpoint vPath, Applied Medical, Rancho Santa Margarita, USA) (7 cm), was inserted through the incision to obtain further access. Pneumoperitoneum is created and standard laparoscopic instruments are used through the Gelpoint.

### Data collection

Data were collected retrospectively from the patients’ files. The following data were retrieved: BMI (Body Mass Index), age at surgery, β-hCG levels before surgery, previous abdominal surgery, previous surgery to the adnexa, previous ectopic pregnancies, parity, smokers/non-smoker, PID, operating time, intra-operative complications, volume of hemoperitoneum, postoperative pain score 6 h after surgery, duration of hospital stay, readmission rates, time to pregnancy after surgery, time to life-born-child after surgery, need for fertility assistance after surgery. Also, data of the first pregnancy after surgery were collected including miscarriage rate, need for induction of labour, gestational age (GA) at birth, mode of delivery and complications during pregnancy. Operating time was collected from the surgical record. Hospital stay was defined as the time from the end of the surgery until discharge from the hospital.

All vNOTES procedures were performed by the same surgeon. The laparoscopic procedures were performed by this surgeon and the other 9 gynaecological surgeons of the hospital. The choice of technique depended on what the surgeon deemed appropriate at the time. The only strict contra-indication for vNOTES surgery was known endometriosis in the pouch of Douglas.

### Statistical analysis

**Fig. 1:**
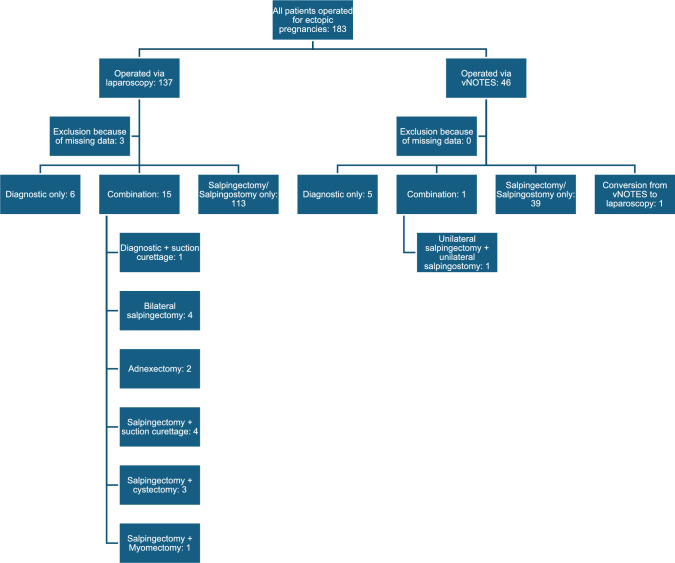
Patient selection

Figure 1 shows the selection process for the two cohorts.

In the laparoscopy group three patients were excluded due to a missing file number and for this reason too much missing data.

In the vNOTES group there was one case that needed conversion to laparoscopy due to lack of good visualisation. This patient remained in the vNOTES group because the intention to treat was the vNOTES approach.

Mann–Whitney *U* tests were used to compare characteristics and outcome measures between the operation methods, vNOTES and laparoscopy.

Chi-square tests, or Fisher Exact tests when numbers were small, were used to compare binary outcome measures.

Ordinal logistic regression was used to compare the duration of hospital stay, expressed as ordinally organised classes, after vNOTES or laparoscopic surgery.

A *p* value less than 0.05 was considered significant. Data were analysed with statistical package SPSS version 27, IBM Corp., Armonk, N.Y., USA.

## Results

Table 1 shows the patient demographics of the two cohorts. No difference was found regarding age, parity, previous abdominal and adnexal surgery, previous PID and volume of hemoperitoneum. The β-hCG level and BMI was significantly lower in the vNOTES group even though the mean difference is small.

**Table 1:** Patient demographics by surgical approach

	vNOTES, *N* = 46			Laparoscopy, *N* = 134			*p* value^a^
	Mean	Min	Max	Mean	Min	Max	
Age (years)	30.2	22	38	30.8	14	43	0.537
BMI (kg/m2)	23.1	16.8	31.6	24.5	17.7	34.9	0.046
β-hCG (IU/L)	3916	23	73,000	3284	29	26,000	0.036
Volume of hemoperitoneum (mL)	367	50	1400	366	50	2000	0.092
	n	(%)		n	(%)		*p* value
Parity > 0	29	(63.0)		70	(52.2)		0.203^b^
Smoking	8	(17.4)		18	(13.4)		0.512^b^
Previous abdominal surgery	19	(41.3)		53	(39.6)		0.841^b^
Previous adnexal surgery	2	(4.3)		19	(14.2)		0.073^c^
Previous EP	1	(2.2)		9	(6.7)		0.245^c^
Previous PID	3	(6.5)		3	(2.2)		0.163^c^

^a^*p* values obtained by Mann–Whitney *U* tests

^b^*p* values obtained by Chi-2 tests

^c^*p* values obtained by Fisher exact tests

*vNOTES* vaginal Natural Orifice Endoscopic Surgery, *Min* minimum, *Max* Maximum, *BMI* Body Mass Index, *β-hCG* beta human chorionic gonadotropin, *EP* ectopic pregnancy, *PID* pelvic inflammatory disease

Table 2 shows the surgical and postoperative results. The operation time using the vNOTES approach was significantly shorter when compared to a laparoscopic approach with a mean surgical time of 35 min compared to 47 min with laparoscopy (*p* < 0.001). All diagnostic procedures and combination surgery was excluded when comparing surgical time as these will influence the surgical time.

**Table 2:** Intra-operative and post-operative results

	vNOTES, *N* = 46			Laparoscopy, *N* = 134			*p* value^a^
	Mean	Min	Max	Mean	Min	Max	
Surgical time (minutes)	34.6	15	60	46.5	15	120	< 0.001
VAS score 6 h postoperaative(resting)	1.9	0	7	2.1	0	6	0.164
VAS score 6 h postoperative (moving)	2.3	0	7	2.8	0	8	0.063
	n	(%)		n	(%)		*p* value^b^
Complications	1			0			
Postoperative infection	0			1			
Duration of hospital stay after surgery							
< 12 h	10	(21.7)	16	(11.9)			0.132
12 h-23	25	(54.3)	76	(56.7)			0.132
24–47 h	8	(17.4)	27	(20.1)			0.132
> = 48 h	3	(6.5)	14	(10.4)			0.132
Not known	0	(0.0)	1	(0.7)			0.132

^a^*p* value obtained by Mann–Whitney *U* Test

^b^*p* value obtained by ordinal logistic regression

*vNOTES* vaginal Natural Orifice Endoscopic Surgery

Both techniques had a very low complication rate. In the vNOTES group one patient had a postoperative bleeding after a vNOTES salpingectomy for which she had a laparoscopic revision.

Both groups had a very low postoperative infection rate. The only patient with an infection (in the laparoscopy group) had an umbilical port site infection that required oral antibiotics. Neither group had a readmission for complications.

Duration of hospital stay was not significantly lower in the vNOTES group but a trend was seen. The duration was calculated from the end of surgery until the time the patient was discharged from the hospital. In the vNOTES group 21.7% of patients left the hospital within the first 12 h after surgery compared to 11.9% in the laparoscopy group. All diagnostic-only and combination surgery was excluded when comparing hospital stay as these can influence the duration of stay.

Both the vNOTES and laparoscopic approach have very low pain scores, measured 6 h postoperatively. All diagnostic-only and combination surgery were excluded for this section as this can influence a patient’s pain score.

In Table 3 and 4 the obstetric outcomes after both vNOTES and laparoscopic surgery are presented. As both groups have a relatively low number of patients, no significant results could be found.

**Table 3:** Obstetric results after surgery

	vNOTES, *N* = 46			Laparoscopy, *N* = 134			*p* value^a^
	*n*	(%)		*n*	(%)		
No pregnancy after EP	10	(21.7)		29	(21.6)		1.000
Pregnancy after EP	36	(78.3)		105	(78.4)		
Baby after EP	29	(63.0)		87	(64.9)		0.823
Type of conception^b^							
Spontaneous	24	(66.7)		69	(65.7)		0.136
IVF	8	(22.2)		28	(26.7)		
Insemination	0	(0.0)		1	(1.0)		
Ovulation induction	4	(11.1)		5	(4.8)		
	Mean	Min	Max	Mean	Min	Max	*p* value^c^
Interval to pregnancy (months)^b^	8.1	0	72	11.2	0	81	0.541

^a^*p* values obtained by Chi-2 tests

^b^Includes women with pregnancies after ectopic pregnancy only

^c^*p* values obtained by Mann–Whitney *U* tests

*vNOTES* vaginal Natural Orifice Endoscopic Surgery, *EP* ectopic pregnancy, *IVF* In vitro fertilisation

**Table 4:** Obstetric results after surgery, women who delivered only

	vNOTES, N = 29			Laparoscopy, N = 87			p value
	n	(%)		n	(%)		
Start of delivery							
Spontaneous start	14	(48.3)		45	(51.7)		0.393^a^
Induction	10	(34.5)		33	(37.9)		
CS	5	(17.2)		7	(8.0)		
Delivery mode							
Vaginal, non instrumental	17	(58.6)		56	(64.4)		0.415^a^
Elective CS	5	(17.2)		6	(6.9)		
Emergency CS	3	(10.3)		8	(9.2)		
Instrumental	4	(13.8)		16	(18.4)		
Perineal tears of patients with vaginal birth							
No tear	3	(13.6)		3	(5.0)		0.2554^a^
Grade I	5	(22.7)		23	(38.3)		
Grade 2	1	(4.5)		8	(13.3)		
Grade 3–4	0	(0.0)		1	(1.7)		
Episotomy	13	(59.1)		25	(41.7)		
Child outcome							
GA < 37w	2	(6.9)		5	(5.7)		0.799^b^
Apgar 1 < 4	0	(0.0)		2	(2.3)		0.561^b^
Apgar 5 < 7	0	(0.0)		3	(3.4)		0.836^b^
Birth weight (g)	Mean	Min	Max	Mean	Min	Max	
	3231	2260	4035	3391	2130	4895	0.119^c^

^a^*p* values obtained by Chi-2 tests

^b^*p* values obtained by Fisher exact tests

^c^*p* values obtained by Mann–Whitney *U* tests

*vNOTES* vaginal Natural Orifice Endoscopic Surgery, *CS* Cesarian section, *GA* Gestational age

In these trends we can see that the vNOTES approach did not increase the risk of miscarriage, did not lengthen the interval to a live born child, did not increase the need for an induction of labour or emergency caesarean section and did not increase the rate of 3rd or 4th degree tears. Patients reached a similar GA at delivery. There was a similar need for tocolysis (Atosiban was used for tocolysis, progesterone only was not included in this group).

## Discussion

In our retrospective cohort study we compare surgical and obstetrical outcome between vNOTES and laparoscopic surgery for extrauterine pregnancies.

This study shows a trend that the duration of hospital stay was shorter with the vNOTES approach compared to laparoscopy. This is an important factor in patient satisfaction and cost. Unless longer observation was required, patients were always given the option to be discharged the same day or to stay the night in the hospital. This is a result that has been observed in different studies that compared vNOTES versus laparoscopy before [7, 10, 15, 16]. A vaginal approach has been found to give less pain than entering the abdomen via the abdominal wall, confirmed by multiple previous publications [7, 11–13, 15–18]. In our study, even though there was a trend, this result was not significant.

Surgical time was significantly lower with a vNOTES approach compared to a laparoscopic approach. Previous studies have shown shorter duration of surgery for vNOTES adnexal surgery and hysterectomy compared to a laparoscopic approach. The shorter surgical time for vNOTES salpingectomy for extrauterine pregnancies could be attributed to the fact that the hemoperitoneum can be evacuated quickly by opening the pouch of Douglas and evacuating the blood manually with a swab whilst the patient is tilted in anti-Trendelenburg. With a laparoscopic approach a suction device is used, that easily gets blocked by blood clots. All surgeries in the vNOTES group were performed by the same surgeon which can also decrease operating time. This shorter surgical time is however consistent in multiple previous studies [10, 12, 13, 15–18].

In the vNOTES group there was one patient that needed conversion to laparoscopy due to poor visualisation after opening of the pouch of Douglas. This case was in the beginning of the surgeon’s learning curve. All other vNOTES surgeries were finished without making an abdominal incision. In patients with known endometriosis, vNOTES is contraindicated.

Overall there were no significant differences between the standard laparoscopic approach and the newer vNOTES approach in terms of complications or pain score. This was seen in previous studies as well [7, 8, 11]. For this reason we can conclude that vNOTES is a good and safe alternative for trained surgeons in the treatment of ectopic pregnancies.

A theoretical concern after performing a vNOTES salpingectomy is that the scar, after the posterior colpotomy, predisposes to extensive vaginal laceration or even rupture during subsequent labour. In our cohort, we could not show any ruptures of the vagina and there was not an increased incidence of any grade of vaginal laceration or sphincter injury. In analogy, Tavano et al. showed that previous vNOTES surgery did not change the mode of delivery for future pregnancies and was not associated with pregnancy related complications [14]. Furthermore, Berisha et al. analysed a cohort of 111 vNOTES adnexal cases and found a very low risk of future dyspareunia [19].

The benefits of a vNOTES approach is that it is a fully vaginal, minimally invasive, scarless approach, with an efficient way to evacuate hemoperitoneum, with no risk of future abdominal wall complications such as hernia. The potential downsides of a vNOTES approach are complications arising with the posterior colpotomy, mainly rectal injury. The cohort by Berisha et al., showed no serious intraoperative complications [19].

A large complications series of a 1000 patients by Baekelandt et al. showed that there was a 1.4% intra-operative complication rate in the hysterectomy group (of which 1.2% was cystotomies) and no intra-operative complications in to non-hysterectomy group. The postoperative complication rate was 3.8% in the hysterectomy group and 0.4% in the non-hysterectomy group (1 patient with a urinary tract infection out of 270 included patients). The conversion rate was 0.4% [20].

The strength of this study is that it is the largest cohort comparing two surgical techniques: laparoscopy versus vNOTES for extrauterine pregnancies with obstetrical data.

The weaknesses of the study include that only one surgeon performed all vNOTES cases versus multiple surgeons in the laparoscopy group. We do not anticipate that there should be any differences in obstetrical outcome depending on mode of surgery for EP, regardless, we do not have enough included patients to have significant results about future pregnancies. This early data however shows promising trends that vNOTES is as safe as laparoscopy for future pregnancies. This confirms the result that was found by Tavano et all in an earlier study [14].

## Conclusion

This study shows that the vNOTES approach is a good and safe technique to operate ectopic pregnancies and can provide shorter operating time and duration of hospitalisation. Early data shows promising results on safety for future pregnancies however these results were not significant because the population was too small. Further research about this topic is warranted.

## Data Availability

No datasets were generated or analysed during the current study.
